# White Matter Tracts and Diffuse Lower-Grade Gliomas: The Pivotal Role of Myelin Plasticity in the Tumor Pathogenesis, Infiltration Patterns, Functional Consequences and Therapeutic Management

**DOI:** 10.3389/fonc.2022.855587

**Published:** 2022-03-02

**Authors:** Hugues Duffau

**Affiliations:** ^1^ Department of Neurosurgery, Gui de Chauliac Hospital, Montpellier University Medical Center, Montpellier, France; ^2^ Team “Plasticity of Central Nervous System, Stem Cells and Glial Tumors”, Institute of Functional Genomics, National Institute for Health and Medical Research (INSERM) U1191, University of Montpellier, Montpellier, France

**Keywords:** brain mapping, cognition, lower-grade glioma, myelin, neural networks, neuroplasticity, white matter tracts, brain connectome

## Abstract

For many decades, interactions between diffuse lower-grade glioma (LGG) and brain connectome were neglected. However, the neoplasm progression is intimately linked to its environment, especially the white matter (WM) tracts and their myelin status. First, while the etiopathogenesis of LGG is unclear, this tumor seems to appear during the adolescence, and it is mostly located within anterior and associative cerebral areas. Because these structures correspond to those which were myelinated later in the brain maturation process, WM myelination could play a role in the development of LGG. Second, WM fibers and the myelin characteristics also participate in LGG diffusion, since glioma cells migrate along the subcortical pathways, especially when exhibiting a demyelinated phenotype, which may result in a large invasion of the parenchyma. Third, such a migratory pattern can induce functional (neurological, cognitive and behavioral) disturbances, because myelinated WM tracts represent the main limitation of neuroplastic potential. These parameters are critical for tailoring an individualized therapeutic strategy, both (i) regarding the timing of active treatment(s) which must be proposed earlier, before a too wide glioma infiltration along the WM bundles, (ii) and regarding the anatomic extent of surgical resection and irradiation, which should take account of the subcortical connectivity. Therefore, the new science of connectomics must be integrated in LGG management, based upon an improved understanding of the interplay across glioma dissemination within WM and reactional neural networks reconfiguration, in order to optimize long-term oncological and functional outcomes. To this end, mechanisms of activity-dependent myelin plasticity should be better investigated.

## Introduction

For many decades, oncological considerations prevailed in the investigation and treatment of brain tumors, whereas the central nervous system *per se* received less attention. However, cerebral neoplasms and their environment, i.e., neural networks, are intimately linked, especially in diffuse tumors such as lower-grade gliomas (LGG) (1–3). Understanding these interdependencies is critical. In neurosciences, recent advances in the field of connectomics have emphasized the pivotal role of the white matter (WM) tracts in cognition and behavior (4, 5). Therefore, the dynamic interplay between WM fibers and LGG should be more systematically explored in order to better predict both the tumor progression and the reactional brain circuitry reconfiguration, with the ultimate aim of tailoring an optimal multistep treatment strategy for each patient.

Here, the goal is to review the implications of constant interactions between WM tracts, with special attention to their myelin status, and LGG concerning (i) the origins of this tumor (ii) the patterns of dissemination of LGG within the cerebral parenchyma (iii) the functional consequences of glioma infiltration (iv) the personalized management to be continuously adapted accordingly. To this end, only diffuse LGG have been studied, by excluding circumscribed gliomas such as pilocytic astrocytoma, pleomorphic xanthoastrocytoma or ganglioglioma.

## Spatio-Temporal Pattern of Myelination During Brain Development and LGG Pathogenesis

Although the causative factors of LGG are still poorly known, some data about their temporal and spatial origins have been reported (6). Regarding its temporal origin, this tumor seems to appear during the adolescence (7). Since the LGG velocity of growth is linear during the initial stage of the disease (8, 9), it was possible to extrapolate backward in time using computational models (especially in incidental LGG) and to estimate glioma date of birth in teenage-hood/early adulthood (7, 10). Concerning its brain spatial distribution, LGG is mostly localized within anterior cerebral regions (11). Thanks to a method of graph-based spatial position mapping (12), a probabilistic atlas of LGG locations revealed a preferential distribution within frontal (33%), insular (37%) and temporal (18%) areas, with very few LGG involving posterior structures (13) – less than 2% of occipital LGG (14–16).

Interestingly, a parallel can be made with the spatiotemporal pattern of myelination, which is a dynamic process in the developing brain and which represents an excellent marker of cerebral maturation (17). Advances in MRI, particularly in diffusion tensor imaging (DTI), showed that WM myelination occurs during ontogeny in highly orderly and predictable patterns (18–20). Regarding its time course, whereas this process is faster during the first decade of life, WM continues to mature during adolescence (21). Concerning its spatial distribution, myelination varies across cerebral regions, with a progression from the posterior to the anterior parts of the brain (22, 23). Especially, myelination occurs earlier in sensory pathways (somatosensory, vision) (24), whereas an increase in the degree of myelination is detectable in the frontal subcortical WM in the late phase of development (25). In addition to this caudo-rostral gradient, myelination occurs earlier in projection tracts than in associative fibers, showing that more complex cerebral structures required for the highest level integrative and executive functions are myelinated later than less complex areas underlying basic neurological functions (26). However, although biologically expensive hubs of the brain connectome were less myelinated than primary cortical areas at 14 years, association areas had faster rates of myelination over the course of adolescence (27). Remarkably, incompletely myelinated axons during teenage-hood and even during young adulthood (28), resulting in variations in conduction velocities within neural circuits, might participate in network-level neuroplasticity through activity-dependent myelination in response to environmental stimuli (29, 30).

Therefore, one could hypothesize that WM maturation process might play a role in the genesis of LGG. Indeed, myelination pattern shows spatiotemporal similarities with the natural history of LGG, i.e., occurring during adolescence, with a predominance of tumor location in regions which have been myelinated later - while LGG rarely involve the sensory areas myelinated earlier. This is in agreement with the retrogenesis hypothesis, based upon changes in WM properties in developing and aging brain, which postulates that late maturating tissue, especially late myelinated axons constituting the “top of the pyramid”, are more vulnerable to decline over the lifespan (31). Furthermore, myelin structural and functional adaptive changes induced by neuronal activity (32), especially in regions involved in higher brain functions such as the prefrontal cortex (33), are underpinned by molecular mechanisms which include modifications in oligodendrocyte precursor cells (OPC) proliferation (34). Importantly, neural regulation of brain development and cancer seem to share similar mechanisms (35). Taken into account the robust mitogenic effect of this neuronal activity on OPC lineage, dysregulation of activity-dependent proliferation signals might contribute to the initiation or growth of brain tumors that molecularly resemble OPCs (36). In reciprocity to this neuronal-activity induced proliferation of tumoral cells (37), gliomas themselves can increase the excitability of the surrounding neural circuits (38). These bidirectional mechanisms of neuron-glial interactions could participate in activity-regulated myelin plasticity (39). This might explain why LGG incidence is elevated in association regions with a high functional connectivity (40). These neural hubs which seem more vulnerable to LGG correspond to brain areas populated with presumed cells of origin for gliomas, especially OPCs, as evidenced by a recent probabilistic map (41). This atlas also showed that gliomas predominantly involved cerebral regions enriched with expression of genes associated with chromatin organization and synaptic signalling, making a link between genetic, cellular and connectomic levels (41). Correlations between molecular profile and glioma location have also been evidenced in oligodendroglial tumors, suggesting that subtypes of oligodendrogliomas may derive from site-specific precursors (42). Furthermore, in a genome-wide association study of diffusion MRI data aiming of exploring genetic variation influencing WM microstructure, among the 25 reported genetic risk regions of glioma, 11 were also correlated with WM microstructure: these findings support the close genetic relationship between glioma and WM integrity (43).

To conclude, parallel mechanisms in both normal myelin plasticity and in glioma have been evidenced, such as the parallel importance of the PI3K/AKT/mTOR pathway [for a review, see (39)]. The implication of these ultrastructural mechanisms in neural networks compensation before and after surgery of LGG have to be explored more, especially by investigating their actual role in the redistribution of the functional connectivity already demonstrated at a macroscopical level (3).

## WM Tracts and Patterns of Glioma Diffusion: The Role of Myelin Characteristics

Since the seminal works by Scherer in 1938 (44), glioma cells are known to migrate along the WM fibers, even at early stages of disease (45, 46). DTI studies evidenced an anisotropic dissemination of LGG with a tropism for main subcortical bundles, such as the pyramidal tract (47), uncinate fasciculus (UF), inferior fronto-occipital fasciculus (IFOF), arcuate fasciculus (AF) (48–50), or the corpus callosum, leading to bilateral invasion (51). Such patterns of tumor diffusion within the fibers tracts resulted on the proposal of new classification systems to distinguish various LGG according to their WM invasiveness, for example for insular/paralimbic gliomas (52). Determination of tumor migration fingerprint within the connectome is essential for adapting a personalized therapeutic strategy, especially for surgical planning [(53), see below].

Nevertheless, mechanisms underpinning glioma invasion are still unclear, even though the need to better understand intercommunications across tumoral and neuronal cells is now emphasized to explain the spatial anisotropy of diffusion (54, 55). Upregulation of genes involved in cell motility might facilitate the spread of both LGG and high-grade gliomas along WM tracts and might contribute to their invasive phenotype (56). Interestingly, the myelin status seems to play a pivotal role in this tumoral dissemination. Indeed, glioma cells migrated along the outer surface of myelin sheaths and/or along neuronal axons inside myelin sheaths (56). Molecules at the level of this myelin sheath may inhibit glioma cell migration and proliferation (46, 57). WM is a pro-differentiative niche for glioblastomas, since glioma cells in contact with WM can acquire pre-oligodendrocyte fate, leading to a decreased proliferation and invasion (58). However, the neoplasm itself may damage WM, especially by secreting mettaloproteinases able to overcome the inhibitory effect of myelin and to create suitable conditions for tumor cell invasion (59). Moreover, Notch pathway activation could represent an important driving force by which glioma cells migrate within WM tracts (46). These mechanisms may explain why glioma cells are mainly distributed along WM fibers, particularly which exhibit a demyelineated phenotype: glioma cells could be more likely to migrate along the surface of unmyelinated axons or to enter axons for invasion *via* unmyelinated regions, i.e., when the myelin sheath was damaged by the neoplasm (46). Indeed, extensive demyelination changes are frequent in WM tracts invaded by glioma, as confirmed by DTI (49, 50).

In summary, it seems that myelin constitutes a protection against glioma cells migration, but that its destruction results in fragility sites facilitating tumor invasiveness. This hypothesis is in accordance with the preferential spatial distribution of LGG previously discussed, namely, in brain locations which were myelinated later.

## WM Tracts as a Main Limitation of Neuroplasticity: Functional Consequences in LGG Patients

Even though LGG frequently involve highly connected functional hubs, patients usually exhibit no or only mild neurological disturbances at diagnosis, due to mechanisms of neuroplasticity progressively induced by this slow-growing neoplasm (2, 60). A recent meta-networking theory of brain functioning revealed a dynamic organization of the central nervous system, with perpetual succession of new equilibrium states relying on constant changes within and between neural networks, and allowing behavioral adaption to the environment as well as reactional reshaping after brain lesion (61). This flexible model breaking with the rigid localizationist dogma explains how functional compensation is possible despite large tumoral infiltration of cerebral areas traditionally conceived as “eloquent” (3). Nonetheless, the neuroplastic potential is not infinite: the WM connectivity represents a major limitation of network reconfiguration, as evidenced by atlases of cortico-subcortical circuits critical for brain functions identified by intraoperative electrostimulation in LGG patients who underwent awake surgery (5, 62, 63). While anatomo-functional variability and plasticity are high at the cortical level, they are very low at the level of the WM tracts (64).

For many years, WM was conceived as electrical wires allowing a simple conduction of information: in fact, a complex transport system with active computational properties has recently been acknowledged (65, 66). Such a neural computing dynamically performed by the WM fibers themselves is highly depending on the myelination status, since myelin around axons facilitates saltatory neurotransmission and affects velocity of action potentials (67). Importantly, myelin remodeling is a continuous process throughout the lifespan (68), which depends on experience (69), i.e., on the acquisition of complex behaviors (70). For instance, learning piano playing (71) or juggling (72) is correlated to the enhancement of WM microstructure in networks underlying the new skill. Activity-dependent myelin regulation can be considered as an additional form of neural plasticity, able to modulate spike time arrival and coordinate neural circuit oscillations (29, 30, 33, 73). Thus, dysregulation of myelin plasticity can have a negative influence on neural processing by disrupting signal integration, propagation and synchronization within and across networks (39). This alteration in neural coherence may have direct clinical impact in brain-damaged patients, e.g., changes in myelin status in patients with multiple sclerosis were correlated with walking difficulty (74).

**Figure 1:**
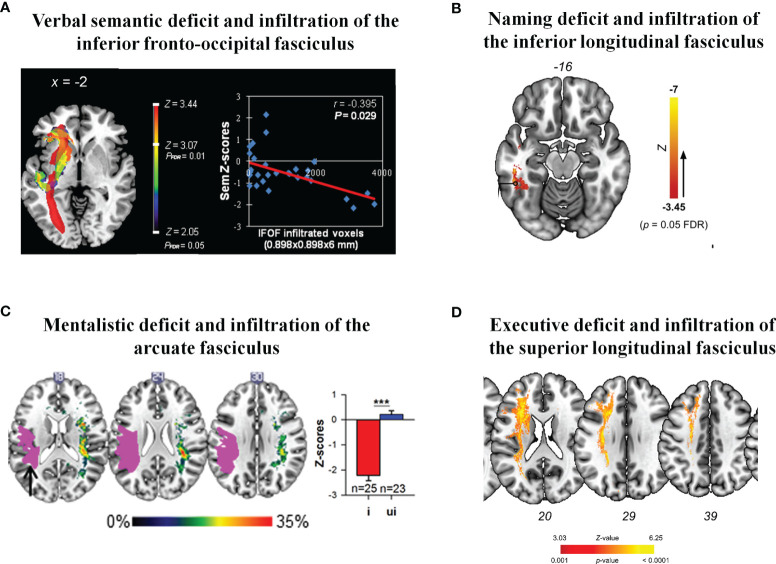
Correlations between the degree of infiltration of WM tracts by the LGG and cognitive deteriorations: **(A)** Preoperative voxel-based lesion symptom map for semantic fluency, evidencing significant relationship between the inferior fronto-occipital fasciculus infiltrated voxels by LGG and deficit of language semantics [from (78) with permission]; **(B)** Voxel-based lesion symptom of postsurgical lasting anomia performed on residual infiltration map, showing correlations between postoperative lexical retrieval troubles and LGG invasion of the left inferior longitudinal fasciculus [from (79) with permission]; **(C)** Significant relationship between postoperative residual tumor volume in the arcuate fasciculus and decreased low-level mentalizing accuracy [from (80) with permission]; **(D)** Disconnectome analysis demonstrating a significant link between postoperative persistent deficit of executive functions and the residual LGG infiltration volume in the superior longitudinal fasciculus [from (81) with permission]. ***Statistically significant.

Therefore, LGG migration within WM fibers can generate functional consequences, partly due to a loss of activity-regulated myelination. When the plastic compensatory capacity is overwhelmed because of glioma-induced WM damage, epileptic activity may occur, which usually leads to the diagnosis of LGG (75). Even though these patients are frequently enjoying an active life, if an extensive neuropsychological examination is performed before any treatment, over 55% of them already experience cognitive impairments (76). By calculating the degree of disconnection of each associative pathway (based upon the degree of LGG infiltration) using voxel-based and tractwise lesion-symptom analyses (77), significant relationships were found between WM tracts invasion and performance decrease in specific domains related to the function subserved by the network invaded. Typically, LGG patients with involvement of the left IFOF may exhibit a decline of semantic fluency at diagnosis (78) (**Figure 1A**). Using each patients’ DTI, reduced fractional anisotropy values in the right superior longitudinal fasciculus (SLF) affected by the glioma were associated to visuospatial impairments (82).

Tractwise and disconnectome-behavior analyses were also performed after LGG surgery in order to correlate postoperative neurocognitive scores to the residual tumor infiltration within the WM fibers - voluntary left for preventing severe long-lasting deficit thanks to a connectome-based resection in awake patients (83). Lexical retrieval impairments were predicted by postsurgical residual lesion volume in the left inferior longitudinal fasciculus (ILF) (79). A deterioration of theory of mind (i.e., low-level face-based mentalizing or empathy) was linked to the degree of disconnection by the residual tumor in the right AF (80, 84), whereas high-level mentalizing capacity (i.e., the ability to infer the intention of other’s) was linked to the residual infiltration in the cingulate fasciculus (80). Some degree of postoperative anosognosia was associated with remaining tumor infiltration in the right cingulate bundle (85). Postsurgical worsening of executive functions was linked to residual glioma invasion within the frontoparietal connectivity, especially with significant correlations between decline of mental flexibility and involvement of the layer II of the left SLF (81) (**Figures 1B–D**).

To sum up, despite some potential of WM plasticity, axonal and myelin-induced injury due to glioma migration may result in seizure and performance decline in LGG patients, which should be objectively assessed by a neuropsychological evaluation before to treat, and which represents a valuable parameter in order to predict the risk of persistent cognitive worsening, particularly following surgical resection. It is worth noting that the brain parenchyma infiltrated by LGG is thought to be more extensive than the FLAIR hypersignal (86). Thus, the real invasion into WM may be underestimated. To improve the sensitivity of neuroimaging, quantitative analysis of DTI indices may provide useful information for assessing tumor microstructures and glioma cell invasion within the WM (86, 87). Indeed, DTI values such as fractional anisotropy and perpendicular diffusivity seem to be sensitive and specific biomarkers, reflecting the integrity of the myelin in various pathological or physiological processes, e.g., WM maturation, demyelination, or dysmyelination (87–89). However, even though these data are promising, it should be acknowledged that they have been acquired in animal models. Therefore, further studies using each patients’ DTI are needed in order to examine the relationship between WM invasion by the glioma, the consequence on myelin, and brain functions.

## Towards More Consideration of WM Tracts For an Adapted Management of LGG Patients

WM infiltration is critical for elaborating an individualized management, both regarding the timing of active treatment(s) which should be proposed earlier (including in incidental LGG), namely, before a too wide involvement of the WM bundles, as well as regarding the anatomic extent of surgical resection and/or irradiation which should take account of the subcortical connectivity (53).

Although the risk of severe persistent neurological deficit is almost nil in recent surgical series using awake mapping for LGG resections, with a high rate (94% to 97%) of return to work (76, 90), extensive postsurgical neuropsychological assessments have nonetheless revealed a subset of patients who kept some degrees of impairment regarding higher-order cognitive functions as well as behavior and personality (76, 85). By using tractwise and disconnectome-behavior analyses, these subtle but objective deficits, which may have a negative impact on quality of life, have mostly been linked to a surgical disconnection of WM fibers. For example, correlations were demonstrated between damage of the left SLF as well as the left frontal aslant tract and lasting executive decline (81); injury of the left UF and heightened schizotypal traits (91); disruption of the left IFOF and behavior changes such as hyperactivity (85); disconnection of the right UF as well as the right IFOF and subjective empathy impairment (92); damage of the right AF and social cognition (mentalizing) deterioration (84); lesion of the left ILF and lexical access disturbances (79); or surgical disruption of the SLF/cingulate bundle and diminished performance in the voluntary deployment of visuospatial attention (93).

These findings confirming the low plastic capacity of the WM fibers play a critical role in the surgical strategy, not only regarding the principle of connectome-based resection relying on the mapping of cortico-subcortical networks critical for brain functions (with special emphasis on the preservation of WM connections) (94), but also concerning the indications of potential reoperation(s) (95). Indeed, the degree of additional functional reorganization occurring after the first surgery and making (or not) possible subsequent resection(s) is constrained by the prominent LGG relapse within the subcortical connectivity (96). Because glioma stem cells are preferentially located along WM fibers exhibiting a demyelinated phenotype at the invasive frontier of tumor tissues (46), the more the neoplasm will exhibit a migratory pattern (rather than a proliferative, bulky one), the less other(s) radical resection(s) will be conceivable for functional issues (53).

Similarly, the neural connectivity should be taken into consideration for adjuvant medical treatments, especially by incorporating WM tracts as structures at risk for planning radiotherapy (97). Delayed radiation-induced cognitive deteriorations are frequent in long-term survivors with LGG (98), mostly due to injury of the WM bundles, as evidenced by correlations between behavior outcomes and DTI following radiotherapy (99–101). For instance, attention and processing speed decline were observed after radiotherapy of the corpus callosum and intrahemispheric WM fibers (102); language deterioration after radiotherapy of left-sided perisylvian WM (103); memory decline after radiotherapy of medial temporal WM (104); or executive function impairment following radiotherapy of the anterior cingulate bundle (105). This progressive disruption of the WM integrity, which occurs even after focal radiation (106), is mainly elicited by axonal degeneration and demyelination (107–109). This was confirmed by DTI studies which showed increased radial diffusion (100, 108), a radiological marker associated with histologic evidence of demyelination (110).

Furthermore, regarding mechanisms underlying the “chemo-brain” phenomenon, namely, chemotherapy-related cognitive impairment (111, 112), an experimental mouse model showed that these neuropsychological effects may be due to depletion of white matter OPC (113), with a block of activity-regulated myelination induced by methotrexate (114).

## Conclusions and Perspectives

While neglected for a long time, WM tracts are of utmost importance in glioma patients, since their infiltration is one of the main causes of poor outcome. From an oncological perspective, a more extensive glioma involvement of WM fibers was correlated to tumor relapse (115), decreased progression-free survival and shorter overall survival (116). From a functional perspective, glioma diffusion along WM pathways which represent the skeleton of the “minimal common brain” (with a low potential of neuroplasticity) (5, 62), is linked to a higher risk of cognitive decline, partly due to a deficit in activity-dependent myelination (39). Such a migratory pattern within the subcortical connectivity should lead to adapt the therapeutic strategy, by tailoring “à la carte” both the surgical resection according to functional boundaries mapped in awake patients as well as the irradiation planning (79, 97). Therefore, incorporation of these connectomal constraints is critical in the quest for optimization of the onco-functional balance *via* individualized multistage management of LGG patients, especially by proposing earlier treatment(s) before a too large diffusion of tumoral cells (53). In this sprit, neurooncologists must refine their understanding of activity-dependent myelin plasticity regulated by oligodendrocyte/OPC dynamics (69, 117, 118), which seems to be a cornerstone in LGG origin and dissemination. To this end, recent models of 3D anisotropic migration have been elaborated (119, 120), which could be helpful to identify new therapeutic targets in order to inhibit glioma invasion along WM (46, 48, 120, 121). Another promising treatment avenue would be to promote remyelination (122), which might result in possible cognitive improvement, as shown in animal model of chemo-brain (114).

## Author Contributions

The author confirms being the sole contributor of this work and has approved it for publication.

## Conflict of Interest

The author declares that the research was conducted in the absence of any commercial or financial relationships that could be construed as a potential conflict of interest.

## Publisher’s Note

All claims expressed in this article are solely those of the authors and do not necessarily represent those of their affiliated organizations, or those of the publisher, the editors and the reviewers. Any product that may be evaluated in this article, or claim that may be made by its manufacturer, is not guaranteed or endorsed by the publisher.
